# SINGLE ANASTOMOSIS GASTRIC BYPASS (ONE ANASTOMOSIS GASTRIC BYPASS OR MINI GASTRIC BYPASS): THE EXPERIENCE WITH BILLROTH II MUST BE CONSIDERED AND IS A CHALLENGE FOR THE NEXT YEARS

**DOI:** 10.1590/0102-6720201700040010

**Published:** 2017

**Authors:** Italo BRAGHETTO, Attila CSENDES

**Affiliations:** 1 Department of Surgery, Faculty of Medicine, University of Chile, Hospital José Joaquin Aguirre, Santiago, Chile

**Keywords:** Single Anastomosis, Gastric bypass, Bile reflux., Anastomose única, Bypass gástrico, Refluxo biliar.

## Abstract

***Introduction*:**

Single anastomosis gastric bypass (one anastomosis gastric bypass or mini-gastric bypass) has been presented as an option of surgical treatment for obese patients in order to reduce operation time and avoiding eventual postoperative complications after Roux-en-Y gastric bypass.The main late complication could be related to bile reflux.

***Aim*:**

To report the experiences published after Billroth II anastomosis and its adverse effects regarding symptoms and damage on the gastric and esophageal mucosa***.***

***Method*:**

For data recollection Medline, Pubmed, Scielo and Cochrane database were accessed, giving a total of 168 papers being chosen 57 of them.

***Results*:**

According the reported results during open era surgery for peptic disease and more recent results for gastric cancer surgery, bile reflux and its consequences are more frequent after Billroth II operation compared to Roux-en-Y gastrojejunal anastomosis.

***Conclusion*:**

These findings must be considered for the indication of bariatric surgery.

## RESUMO

***Introdução*:**

Bypass com anastomose única ou mini-bypass gástrico foi apresentado como oopção de tratamento cirúrgico para pacientes obesos, a fim de reduzir o tempo da operação e evitar complicações pós-operatórias após bypass gástrico Y-de-Roux. A principal complicação tardia pode estar relacionada ao refluxo biliar.

***Objetivo*:**

Relatar as experiências publicadas após a anastomose Billroth II e seus efeitos adversos em relação aos sintomas e danos sobre a mucosa gástrica e esofágica.

***Método*:**

A coleta de dados foi baseada na busca nas bases Medline, Pubmed, Scielo e Cochrane. Um total de 168 artigos foram revisados, tendo sido escolhidos 57 deles.

De acordo com os resultados relatados durante a operação da era aberta para doença péptica e resultados mais recentes para o tratamento cirúrgico do câncer gástrico, o refluxo biliar e suas consequências são mais frequentes após o Billroth II em comparação com a anastomose gastrojejunal em Y-de-Roux.

***Conclusão*:**

Esses achados devem ser considerados para a indicação de cirurgia bariátrica.

## INTRODUCTION

In the last years single anastomosis gastric bypass (SAGB) (one anastomosis gastric bypass or mini-gastric bypass) has been presented as an option of surgical treatment for obese patients in order to reduce operation time and avoiding eventual postoperative complications after Roux-en-Y gastric bypass (RYGBP)^4^ ^,^ ^33^ ^,^ ^37^. Up to now the results with this procedure in terms of weight loss, BMI reduction, and improvement of co-morbidities are quite similar to the RYGBP and sleeve gastrectomy^6^ ^,^ ^30^ ^,^ ^55^. However, a potential risk of complications related to bile reflux is possible, even if modifications of the technique in order to prevent it have been introduced. It is not confirmed whether with these technical modifications we can completely avoid bile reflux. Only clinical results concerning to symptoms or endoscopic findings have been published. Late bile related complications and objective evaluations are missing in order to demonstrate that bile reflux and its consequences do not exist.

The objective of this article is to perform an analysis of the reported experiences with Billroth II (BII) anastomosis and its adverse effects regarding symptoms and objective damage on the gastric and esophageal mucosa in order to consider these problems in patients who will be submitted to SAGB and to promote more objective investigations.

## METHOD

The most relevant literature concerning the experience with BII anastomosis published during the era of peptic ulcer surgery and the more recent publications regarding the results after laparoscopic BII anastomosis after distal gastrectomy for gastric cancer (specially performed in Asian countries) were analysed. For data collection, Medline, Pubmed, Scielo and Cochrane were included. For search publications terms as “bile reflux after Billroth II” “bile reflux” “bile gastritis after gastrectomy” and “gastric stump cancer were used”. A total of 168 papers was reviewed choosing 57 of them to be included for the analysis focused on the presence of symptoms, effects on the esophageal and gastric mucosa and objective evaluation of bile reflux comparing the results published after BII anastomosis vs. RYGBP reconstruction.

## RESULTS

For restoration of gastrointestinal tract after partial distal resection of the stomach there are three options: gastroduodenostomy (Billroth I), gastrojejunostomy with BII anastomosis or with Roux-en-Y anastomosis. After Billroth I or II there are three potential risks: biliary gastritis, bile esophagitis or gastric cancer which have a close relationship with bile reflux into the gastric stump. Regarding to the presence of bile reflux into the gastric stump and distal esophagus there is enough evidence reported in previous publications during the period of peptic ulcer surgery and also more recent literature after laparoscopic distal gastrectomy for gastric cancer.

**FIGURE 1:**
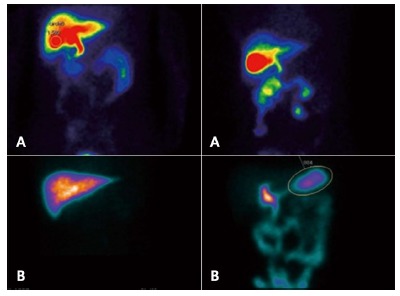
Examination of duodenogastric reflux by ^99m^Tc-ethyl hepatic iminodiacetic acid test: A) negative: B) positive

Bile reflux has been evaluated with scintigraphic assessment - Bilitec2000 -, or bile salt concentration measurement. Figure 1 shows the typical scintigraphic image demonstrating reflux after BII anastomosis and no reflux after Roux-en-Y gastrojejunostomy (RYGJ) anastomosis. Bile reflux has demonstrated to be higher after BII reconstruction compared with RYGJ. High concentrations of bile acid seem to be associated with an elevated risk of intestinal metaplasia. It has been demonstrated a very strict relationship between bile reflux and appearance of symptoms secondary to different grade of gastritis ^3^ ^,^ ^27^ ^,2,^ ^9^.

After gastric surgery due to peptic ulcer disease, 25% of patients have postoperative symptoms, and 5% of them present severe symptoms at the early or middle term follow-up due to esophagitis and Barrett´s or biliary gastritis due to bile reflux. The most frequent symptoms are heartburn, pain, abdominal fullness, early satiety**,** diarrhea or dumping, the last due to fast gastric emptying or small remnant syndrome. The other important late complication is the development of stump gastric cancer mainly associated with bile reflux^5^ ^,^ ^38^ ^,^ ^18^ ^,^ ^45^ ^,^ ^29^ ^,^ ^31^ ^,^ ^25^ ^,^ ^47^ ^,^ ^20^ ^,^ ^57^. 

Since the 80´s decade, there is a hugh evidence concerning the high rate of endoscopic and histological damage at the esophageal or gastric mucosa secondary to bile reflux. D´Amato^12^ published endoscopic and histological reflux esophagitis after BII anastomosis in 47% of patients and only 13% after RYGJ anastomosis and De Vita^21^ demonstrated endoscopic gastritis in 88.8% and histological atrophic gastritis in 94.4% after BII and only 29.4% and 58% of superficial gastritis after Roux-en-Y anastomosis, respectively (p<0.001). Csendes et al.^11^, in a prospective randomized study demonstrated symptoms of gastroesophageal reflux in 33.3% after BII operation compared to 3.2% after RYGJ (p<0.002). In adition, they reported endoscopic esophagitis with intestinal metaplasia at the distal esophagus in 20% of patients after BII, while after RYGJ these findings were present in 3.2% (p<0.001). In the same paper, bile reflux to gastric stump with chronic athrophic gastritis appeared in 40% of cases after BII against 10% after RYGJ^11^. 

More recently, Asian authors^38,18,45,29,31,25,47,20,57^ have published similar results evaluating presence of bile reflux, reflux esophagitis and histologic gastritis after distal gastrectomy for gastric cancer comparing BII vs. RYGJ. They confirmed that erosive esophagitis grade A or B is significantly more frequent after BII anastomosis than after RYGJ (53.6% vs. 23% respectively, p<0.017) and bile reflux and gastritis was present in almost 85% of patients with BII operation. A metanalysis of Zong et al^57^ comparing Billroth I vs. BII vs. Roux-en-Y following distal gastrectomy based on 15 studies, concluded that Billroth I or II reconstruction showed significantly more reflux symptoms, increased gastritis and esophagitis, compared to patients with Roux-en-Y gastrojejunostomy, and quality of life was significantly improved in patients with Roux-en-Y reconstruction. This meta-analysis highlights clinical advantages of the last after distal gastrectomy. 

In Table 1, 2 and 3 comparative results obtained from the literature reviewed in terms of postoperative symptoms of bile reflux and objective endoscopic and histological findings after BII vs. RYGJ are presented. All these studies concluded that BII reconstruction is associated with increased bile reflux in near to 70-80% of patients promoting symptoms, erosive esophagitis, Barrett´s and gastritis^51^ ^,1,^ ^34^ ^,^ ^17^. 

**TABLE 1:** Symptoms related to bile reflux after BII vs. Roux-en-Y gastrojejunostomy (%)

	Billroth II - mean (variation)	Y-de-Roux - mean (variation)
Asymptomatic	45,5 (36-83,3 )	80,6 (74,6- 96,8)
GERD Symptoms	15,5 (10,9-24,4)	7,5 (3,2-17,2)
Gastrointestinal symptoms	12,9 (5,8-23,1)	8,5 (0-25)

**TABLE 2:** Endoscopic findings related to bile reflux after Billroth II and Roux-en-Y gastrojejunostomy (%)

	Billroth II - mean (variation)	Y-de-Roux - mean (variation)
Esophagus		
Normal	56,2 (46,2 70,9)	82,6 (75-90,3)
Erosive esophagitis	30,6 (2,4-53,9)	9,2 (0-25)
Barrett	2,5	0
Carcinoma	3,0	0
Stomach		
Normal	17,9 (4,1-34,2)	78,5 (35-100)
Erosive esophagitis	87,8 (82,3-96,1)	37,3 (17,4-65,1*)
Presence of bile	77,9 (66-88)	16,9 (3,7-42*)

**TABLE 3:** Histologic findings of distal esophagus and gastric stump after Billroth II or Roux-en-Y gastrojejunostomy in patients submitted to distal gastrectomy (%)

	Billroth II - mean (variation)	Y-de-Roux - mean (variation)
Distal esophagus		
Esophagitis	19,3 (2,4-45,4*)	15,9 (0-45,2*)
Intestinal metaplasia	25,6 (21,2-30)	1,6 (0-3,2)
Carcinoma	3,1	0
Gastric stump		
Normal mucosa	15,1	16
Gastritis	71,8 (39,4-94,4* )	14,7(1,8- 58**)
Intestinal metaplasia	6,1	0

**FIGURE 2:**
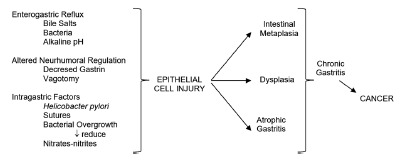
Mechanisms involved in the development of stump gastric cancer after distal gastrectomy

The second important complication is regarding to the risk of developing gastric cancer late after surgery. There is vast information about the pathogenesis and incidence of gastric stump carcinoma. First at all, the pathophysiological mechanisms involved have been studied by several authors. Enterogastric bile reflux induces damage of gastric mucosa, hypochloridia favouring the bacterial colonization and presence of secondary bile acid, all together proved factors for carcinogenesis, developing chronic athrophic gastritis, intestinal metaplasia, inducing adenocystic changes, abnormal cell kinetics; the final result is the appearance of gastric stump carcinoma. In gastric stump, the *Helicobacter pylori* infection rate range 17-68%^39^ ^,^ ^10^ ^,^ ^49^ ^,^ ^46^ ^,^ ^43^ ^,^ ^35^ ^,^ ^15^ ^,^ ^40^ ^,^ ^56^ ^,^ ^23^ (Figure 2).

Definitively, high levels of duodenogastric reflux observed after BII gastric resection are associated with high bile reflux with polyamine concentration in the gastric mucosa, associated with bacterial microflora, presence of mainly free secondary and primary bile acids which may contribute to the high incidence of cancer in the gastric remnant observed after BII operations. All these facts can be considered as important causal factors of the increased risk of gastric stump cancer after this operation^39^ ^,^ ^10^ ^,^ ^49^ ^,^ ^46^ ^,^ ^43^ ^,^ ^35^ ^,^ ^15^ ^,^ ^40^ ^,^ ^56^ ^,^ ^23^. 

Currently due to the improvement of medical treatment with PPI´s inhibitors, gastrectomy for benign disease has decreased over the last two decades. Still, this type of gastric stump carcinoma has not decreased due to the long latency period required for carcinogenesis after initial surgery. Hokosawa and Morgagni^19^ ^,^ ^41^ described the acumulative risk of developing gastric stump cancer after curative distal gastrectomy for early gastric cancer which was 2.5% at 5-years, 2.5 to 6.1% at 10-years, 3.2% at 15-years and 4% at 20-years follow-up ^19,41,44,50,13,26,52,36,54,28,53,21,48^ . The incidence range from 1-8% of patients submitted to distal gastric resection with BII anastomosis. The incidence is 4-7 times more frequent compared to the general population and increases 28% each 5-year follow up. The interval time for the appearance ranges from 4-57 years. The risk decreases depending the age of the initial operation. Patients over 50 years have low risk of developing stump gastric carcinoma, but bariatric patients very often have less than 30 years when are submitted to surgery, and therefore the risk to have cancer at 50 year age is considerable. Table 4 shows the reported prevalence of stump gastric carcinoma after distal gastrectomy exclusively for benign causes^13^ ^,^ ^26^ ^,^ ^52^ ^,^ ^36^ ^,^ ^54^ ^,^ ^28^ ^,^ ^53^ ^,^ ^21^. 

**TABLE 4:** Summary of stump gastric carcinoma after distal gastrectomy for benign disease.

Authors	( ref)	Intake interval (years)
Di Leo	(42)	34,6 (8-57)
Komatsu	(43)	30,0 (4-51)
Tanigawa	(45)	25,8 (10-40)
Lundergath	(46)	20,0 (5-30)
Tersmette	(47)	17,5 (15-20)
Lagergren	(48)	20,5 (15-30)
Tassi	(50)	30,0 (6-42)

Recent publications have reported gastric cancer after non-resectional gastric bypass, situation in which the same pathophysiological mechanism with bile reflux and presence of bacterial colonization can occurs. Orlando et al^44^ published a review of the literature about the cases of gastric cancer arising after any bariatric procedure. Globally, 17 case reports describing 18 patients were retrieved, including the case study by the authors. The diagnosis of tumor was at a mean of 8.6 years after bariatric surgery, 9.3 years after RYGB, and 8.1 years after restrictive procedures. The adenocarcinoma represented most cases localized in the gastric stump (83%). After a restrictive procedure, the cancer was localized in the pouch in 62.5% of cases, in the pylorus in 25%, and in lesser curvature in 12.5%. Scozzari et al^50^ in other review including 28 articles described 33 patients retrieved. Neoplasms were diagnosed at a mean of 8.5 years after bariatric surgery (range two months to 29 years). Node involvement was reported in almost 60% of cases, and distal metastases in 15%. Reported mortality rate was 48.1%. To date, it is not possible to quantify the incidence of esophagogastric cancer after bariatric surgery because of the paucity of reported data. However, being the main concern the delay in diagnosis, it is of critical importance to evaluate carefully any new upper digestive tract symptom appearing after bariatric surgery. Other important point to take in account is the fact that most of the available data are coming from areas with low rate of gastric cancer compared to Asian or Latin-American countries where gastric cancer has a high incidence. 

The study of Inoue et al^21^ shows that RYGB reduces the risk of gastric cancer in an experimental model of dietary-induced carcinogenesis due to lower bile reflux, and a lower bacteria concentration in the gastric content. This data suggest that RYGB may be a safe option for the treatment of morbid obesity even in areas with high gastric cancer incidence. 

## CONCLUSION

In front to these previous experiences and pathophysiologic considerations, SAGB appears as a surgical technique that rapes important surgical concepts. Actually, different authors propose some modifications of classical BII procedure by suturing the jejunum very high along the vertical stapled line in order to avoid bile reflux. Others propose to perform a long and thin gastric tube and they believe that in this way it is possible to create a low pressure system in order to favour the gastric emptying^4^ ^,^ ^33^ ^,^ ^37^ ^,^ ^6^ ^,^ ^30^ ^,^ ^55^. All these mechanisms are conducted to avoid bile reflux. However, gastric physiology is not only a mechanical event^4^ ^,^ ^33^ ^,^ ^37^ ^,^ ^6^ ^,^ ^30^ ^,^ ^55^ ^,^ ^48^ ^,^ ^32^ ^,^ ^42^ ^,^ ^8^ ^,^ ^7^. Up to now in the available literature concerning to SAGB, only clinical studies have been reported, specially focused in the weight loss and improvement of comorbidities. Criticism and prejudice against this procedure was raised by surgeons who preferred a more complex procedure, such as laparoscopic RYGB. Increasing data indicate that the procedure is an effective and durable bariatric procedure. SAGB has lower operation risks compared to RYGB. The weight loss is better after SAGB because of a greater mal-absorptive component than RYGB, but SAGB has a higher incidence of micronutrient deficiencies. Randomized controlled trial and long-term data demonstrated that SAGB can be regarded as a simpler and safer alternative to RYGB. The authors have proposes to renamed “single-anastomosis gastric bypass (SAGB)” because the key feature of SAGB is the “single anastomosis” compared with the two anastomosis of RYGB^54^ ^,^ ^28^ ^,^ ^53^ ^,2148,^ ^32^ ^,^ ^42^. This technique is not exempt of surgical complications, such as Petersen´s internal hernia or afferent loop apart of bile reflux^54^ ^-^ ^57^. Few papers have been dedicated to objective evaluation of bile reflux. Johnson et al ^22^ reported bile reflux in almost 60% of patients similar to the papers published during the era of peptic ulcer surgery^3^ ^,^ ^2^ ^,^ ^22^. We have no information about the endoscopic and histologic damage of esophageal and gastric mucosa, Bilitec 2000, bile salt concentration and type of bile salts at the gastric stump, or scintigraphic assessment for bile reflux now in patients submitted to SAGB. It is necessary to develop these objective studies in order to exclude or confirm the presence of distal esophagus or gastric stump damage. This is the challenge for surgeons interested in demonstrated the advantages of SAGBP during the next years in order to convince that this technique is an option for bariatic surgery without the risk of the complications analyzed. After the results of these studies we can delucidate the controversy.
